# Multidimensional biomarkers for multiple system atrophy: an update and future directions

**DOI:** 10.1186/s40035-023-00370-0

**Published:** 2023-07-28

**Authors:** Linlin Wan, Sudan Zhu, Zhao Chen, Rong Qiu, Beisha Tang, Hong Jiang

**Affiliations:** 1 Department of Neurology, Xiangya Hospital, Central South University, Changsha, 410008 China; 2 Department of Neurology, The Third Xiangya Hospital, Central South University, Changsha, 410013 China; 3 Key Laboratory of Hunan Province in Neurodegenerative Disorders, Central South University, Changsha, 410008 China; 4 National Clinical Research Center for Geriatric Disorders, Xiangya Hospital, Central South University, Changsha, 410008 China; 5 Department of Radiology, Xiangya Hospital, Central South University, Changsha, 410008 China; 6 Hunan International Scientific and Technological Cooperation Base of Neurodegenerative and Neurogenetic Diseases, Changsha, 410008 China; 7 School of Computer Science and Engineering, Central South University, Changsha, 410083 China; 8 National International Collaborative Research Center for Medical Metabolomics, Central South University, Changsha, 410008 China

**Keywords:** Multiple system atrophy, Biomarker, Fluid, Tissue, Gut microbiota, Imaging

## Abstract

Multiple system atrophy (MSA) is a fatal progressive neurodegenerative disease. Biomarkers are urgently required for MSA to improve the diagnostic and prognostic accuracy in clinic and facilitate the development and monitoring of disease-modifying therapies. In recent years, significant research efforts have been made in exploring multidimensional biomarkers for MSA. However, currently few biomarkers are available in clinic. In this review, we systematically summarize the latest advances in multidimensional biomarkers for MSA, including biomarkers in fluids, tissues and gut microbiota as well as imaging biomarkers. Future directions for exploration of novel biomarkers and promotion of implementation in clinic are also discussed.

## Introduction

Multiple system atrophy (MSA) is a rare, rapidly progressing neurodegenerative disease first reported in 1969 [1]. The pathological hallmark of MSA is glial cytoplasmic inclusions (GCIs) consisting of misfolded α-synuclein (α-syn) in affected brain regions, including striatonigral and olivopontocerebellar systems. Due to the presence of α-syn aggregation in the brain, MSA is identified as a synucleinopathy, together with Parkinson's disease (PD) and Dementia with Lewy bodies (DLB). MSA is an orphan disease with an average annual incidence of 0.6–0.7 per 100,000 person-years in Western countries [2]. Clinically, MSA is characterized by various combinations of progressive autonomic failure, cerebellar ataxia, parkinsonian syndrome and pyramidal features. According to the predominant motor symptom, MSA can be divided into the parkinsonian subtype (MSA-P) and the cerebellar subtype (MSA-C). The average survival of MSA patients is 6–10 years from symptom onset to death and very few patients can survive more than 15 years [3]. Currently, there is no effective treatment strategy for MSA.

Based on the Movement Disorder Society Criteria for the diagnosis of MSA, the diagnostic certainty includes four levels: neuropathologically established MSA, clinically established MSA, clinically probable MSA and possible prodromal MSA. The autopsy confirmation is still the gold standard for definite diagnosis of MSA [4]. Because of its clinical heterogeneity and overlapping manifestations with other diseases, including PD, progressive supranuclear palsy (PSP), corticobasal degeneration (CBD), and sporadic adult-onset ataxia of unknown aetiology (SAOA), the clinical diagnosis for MSA is quite challenging [5, 6]. Therefore, biomarkers facilitating the precise diagnosis is necessary in clinical practice. In addition, when evaluating the disease severity and therapeutic effect, the unified multiple system atrophy rating scale (UMSARS) remains the main tool [7]. However, the semiquantitative rating pattern and the ceiling effect have limited its reliability [8]. Thus, there is also an urgent demand for sensitive biomarkers of MSA for monitoring disease severity and therapeutic effects.

**Fig. 1:**
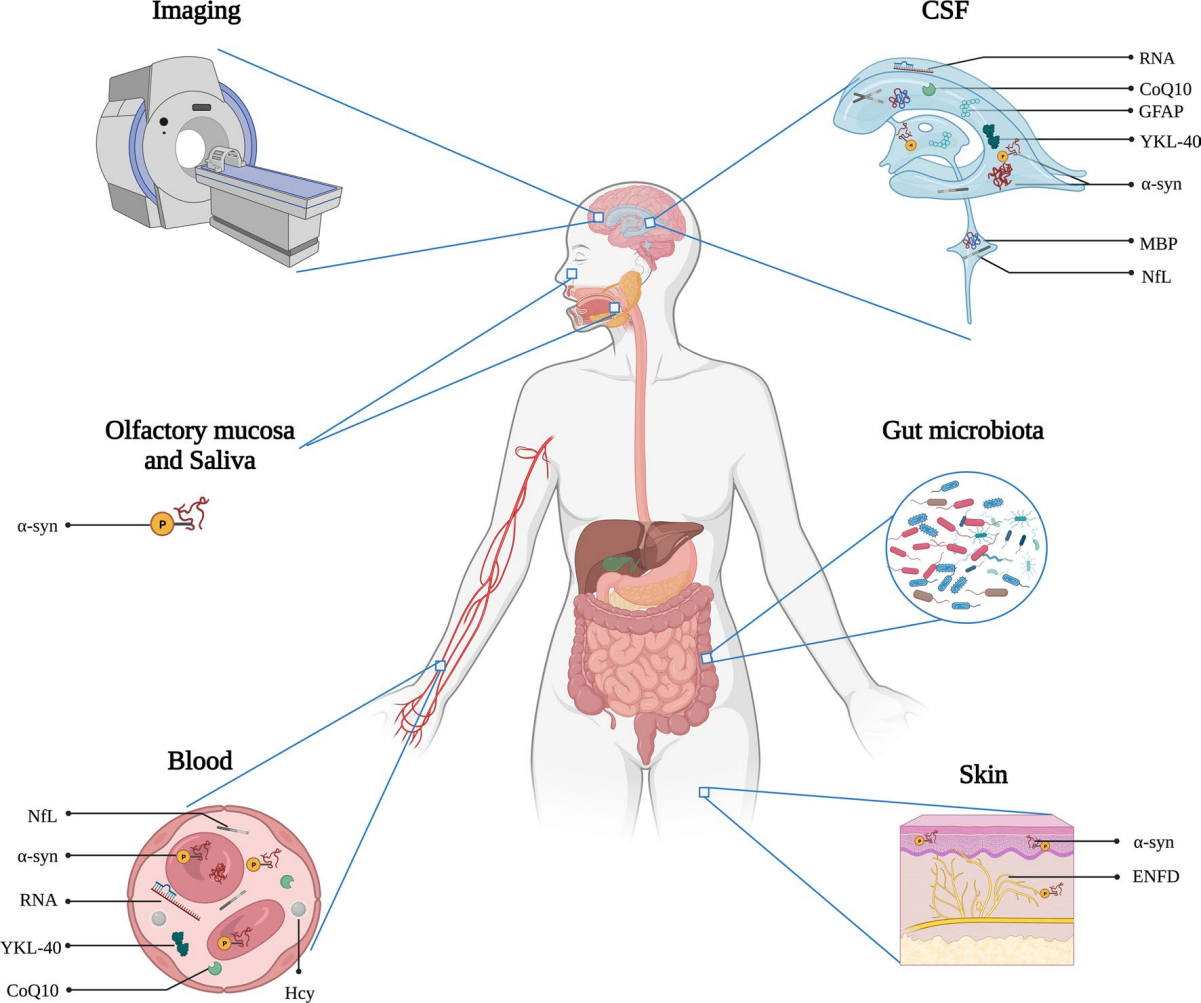
Schematic overview of multidimensional biomarkers for MSA, including fluid, tissue, gut microbiota and imaging biomarkers. Illustration created with BioRender.com

The Biomarker Definitions Working Group has defined a biomarker as “a characteristic that is objectively measured and evaluated as an indicator of normal biological processes, pathogenic processes or pharmacologic response to a therapeutic intervention” [9, 10]. In previous studies of MSA, researchers have explored the potential value of biomarkers from different sample sources such as fluids (including cerebrospinal fluid, saliva, blood and urine), tissues, stool and imaging [11]. However, few biomarkers are available in the clinical practice. In this review, we systematically summarize the multidimensional candidate biomarkers for MSA and discuss their prospects in clinical practice, aiming to provide directions for future research (Fig. 1).

## Fluid biomarkers

### α-Syn

**Fig. 2:**
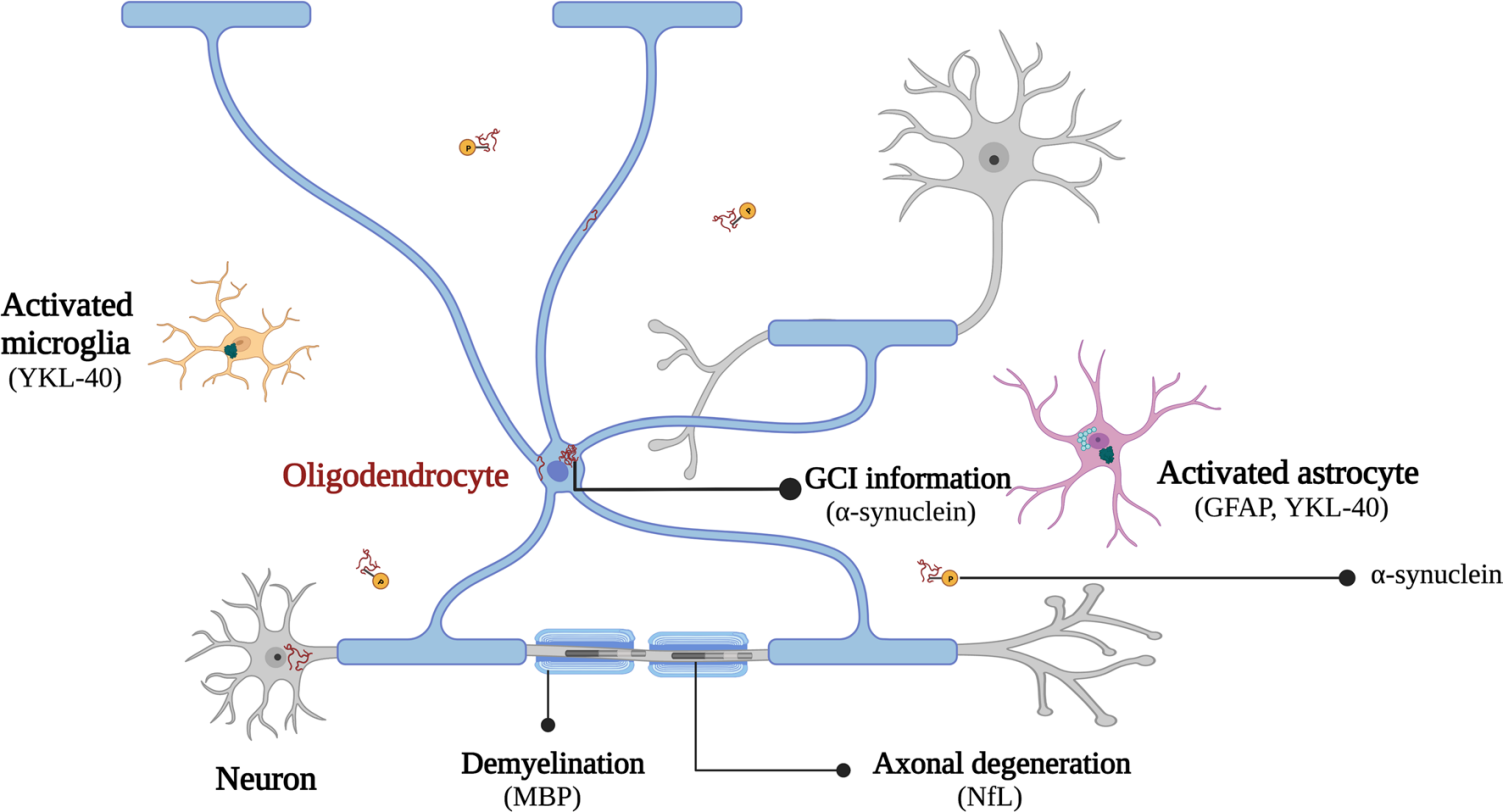
Pathological mechanisms involved in MSA and related biomarkers. Illustration was created with BioRender.com

α-Syn is composed of 140 amino acids and present at presynaptic terminals. The precise function of α-syn is obscure, but currently it is considered as a regulator of neurotransmitter release and synaptic integrity [12]. There are three main forms of α-syn examined in MSA, i.e., total α-syn (t-α-syn), phosphorylated α-syn (pS129-α-syn) and α-syn oligomers (o-α-syn) [13, 14]. Although the deposition of aggregated α-syn in the cytoplasm of oligodendroglia is considered as a pathologic hallmark of MSA (Fig. 2), whether it can serve as a reliable biomarker of MSA is still controversial.

Quite a few researchers have measured α-syn in cerebrospinal fluid (CSF). Most of the studies found lower CSF levels of t-α-syn in MSA patients compared to healthy controls (HCs) [15–22] and three studies found no significant difference [23–25]. One autopsy study showed an increasing tendency of t-α-syn in the postmortem CSF of MSA patients [26]. The conflicting conclusions might be attributed to the neuronal release of α-syn into the CSF and peripheral blood. As for the differences among MSA and other neurodegenerative diseases, some studies illustrated significantly decreased levels of CSF t-α-syn in MSA compared to PSP and CBD [14, 27]. However, the majority of studies found no significant differences of CSF t-α-syn among MSA and other α-synucleinopathies, PSP or CBD [17, 21, 23, 24, 26, 28], suggesting limited value in differential diagnosis (Table 1).

**Table 1:** Potential fluid protein and metabolite biomarkers for MSA

Biomarker	Fluid	Method	MSA versus HC (cohorts)	MSA versus PD (cohorts)	References
T-α-syn	CSF	ELISA BBMA	Decreased (Sweden cohort, UK cohort, Germany cohort, US cohort) Increased (UK cohort) No difference (Netherlands cohort, Japan cohort)	No difference (Germany cohort, US cohort, UK cohort, Netherlands cohort, Japan cohort)	[15–21, 23–26]
	Blood	ELISA	Increased (China cohort, South Korea cohort) No difference (Germany cohort)	Decreased (South Korea cohort) No difference (Germany cohort)	[17, 31, 32]
PS129-α-syn	CSF	ELISA BBMA	Decreased (US cohort) No difference (UK cohort)	Decreased (US cohort) No difference (UK cohort, Greece cohort)	[14, 20, 26]
	RBC	ELISA	Increased (Chinese cohort)	NA	[35]
O-α-syn	CSF	ELISA	No difference (UK cohort)	No difference (UK cohort)	[26]
	RBC	ELISA	Increased (Chinese cohort)	No difference (Chinese cohort)	[33, 34]
CoQ10	CSF	ELISA	Decreased (Spain cohort)	Decreased (Spain cohort)	[59]
	Blood	UPLC-MS HPLC	Decreased (China cohort, Japan cohort)	No difference (China cohort, Japan cohort)	[60–62]
NfL	CSF	ELISA SIMOA	Increased (UK cohort, Germany cohort, Sweden cohort, Netherlands cohort, France cohort, Spain cohort, Russia cohort)	Increased (UK cohort, Germany cohort, Sweden cohort, Netherlands cohort)	[16, 21, 71–74]
	Blood	SIMOA	Increased (UK cohort, Sweden cohort, France cohort, Spain cohort, Germany cohort, Russia cohort, Netherlands cohort)	Increased (UK cohort, Sweden cohort, Netherlands cohort)	[66, 73, 74]
YKL-40	CSF	ELISA	Increased (UK cohort) No difference (Sweden cohort)	Increased (Sweden cohort) No difference (UK cohort)	[16, 79, 80]
	Blood	ELISA	No difference (Sweden cohort)	No difference (Sweden cohort)	[80]
GFAP	CSF	ELISA SIMOA	No difference (US cohort, Germany cohort, Netherlands cohort)	Increased (Germany cohort) No difference (Netherlands cohort)	[82–84]
MBP	CSF	ELISA	Increased (Netherlands cohort)	Increased (Netherlands cohort)	[84, 87]
Hcy	Blood	SPCEI	Increased (China cohorts)	Increased (male) (China cohorts)	[96–99]

*BBMA* bead-based multianalyte assay, *CoQ10* coenzyme Q10, *CSF* cerebrospinal fluid, *ELISA* enzyme-linked immunosorbent assay, *GFAP* glial fibrillary acidic protein, *HC* healthy control, *Hcy* homocysteine, *HPLC* high-performance liquid chromatography, *MBP* myelin basic protein, *MSA* multiple system atrophy, *NA* not available, *NfL* neurofilament light protein, *O-α-syn* oligomeric α-synuclein, *PS129–α-syn* serine 129 phosphorylated α-synuclein, *SIMOA* single molecular array, *SPCEI* solid-phase competitive chemiluminescent enzyme immunoassay, *T-α-syn* total α-synuclein, *UPLC-MS* ultra-performance liquid chromatography coupled with mass spectrometer

Phosphorylation at serine 129 is a common posttranslational modification of α-syn, which is more disease-specific and might improve the toxicity of α-syn by promoting its aggregation [29]. However, some studies in recent years have also implied a potential protective role for pS129-α-syn as it apparently inhibits further aggregation [30]. Although the exact role of pS129-α-syn remains undetermined, its value as a biomarker has been explored. One study found significantly decreased CSF pS129-α-syn levels in MSA than in HC and PD [20]. However, other studies did not replicate the results, showing no remarkable differences of CSF pS129-α-syn between MSA and HC or PD [14, 26]. Moreover, the pS129-α-syn/t-α-syn ratio in the CSF of MSA patients is much higher than that in HC, but does not significantly differ from that in PD patients [14, 20]. Another form of α-syn, oligomeric α-syn (o-α-syn), shows no significantly different postmortem CSF levels among patients with MSA, PD, PSP and HC [26]. Thus, the oligomeric form of α-syn might have limited value as a biomarker for MSA (Table 1).

The values of α-syn levels in blood are also undetermined. Two studies showed significantly higher levels of plasma t-α-syn in MSA patients than in HCs [31, 32], while another study found no significant difference in serum t-α-syn [17]. A meta-analysis reported that the plasma levels of t-α-syn in MSA patients are significantly increased compared with HC; however, the analysis only included two studies [22]. For comparison between MSA and PD, one study demonstrated decreased plasma t-α-syn levels in MSA [31], while another reported no significant difference [17]. In addition, recently, different types of α-syn in red blood cells (RBCs) have been investigated since erythrocytes are the major source of peripheral α-syn. Generally, levels of oligomeric and phosphorylated α-syn in RBCs have been reported to be significantly increased in MSA patients than in HCs [33–35]. One of these studies showed that pS129-α-syn levels in RBCs could distinguish MSA patients from HCs with a sensitivity of 80.37%, a specificity of 88.64% and an area under the curve (AUC) of 0.91, suggesting a high diagnostic value of pS129-α-syn in RBCs for MSA (Table 1). However, the ability of α-syn in RBCs to differentiate MSA from other neurodegenerative diseases including PD, PSP and CBD still need further exploration [35].

Recently, seeding aggregation assays (SAAs), including real-time quaking-induced conversion (RT-QuIC) technology and protein-misfolding cyclic amplification (PMCA), have been applied to measure the seeding activity of pathological α-syn [36–38]. These two assays were originally used for the detection of pathological prion proteins in body fluids and skin tissues [39–41]. Increasing evidence suggests that pathological α-syn spreads throughout the nervous system in a prion-like manner, acting as seeds to induce further aggregation of native α‐syn [42, 43]. Thus, seed amplification assays may be used for the detection of pathological α-syn, even a very small amount of α-syn, by amplifying the abnormal protein to detectable levels [38, 44, 45]. In CSF samples, three studies revealed good performance of seed amplification assays in differentiating MSA from HC [45–47]. However, another two studies found quite low rates of positive RT-QuIC results in MSA patients [48, 49]. This discrepancy may be caused by differences in specific experimental conditions. By contrast, CSF seed amplification assay results consistently showed good performance in differentiating MSA from PD and DLB, suggesting the presence of different conformational strains of α-synuclein among α-synucleinopathies [45–49]. The correlation analysis showed that all RT-QuIC parameters were associated with worse clinical progression of MSA [47] (Table 2).

**Table 2:** Seeding activity of pathological α-syn in different samples from MSA patients measured by seeding aggregation assays

Sample	Research group	Participants	Assay(s)	Main findings
Cerebrospinal fluid	Singer et al. [46]	62 MSA, 16 PD, 13 DLB, 29 HC	PMCA	96.8% (60/62) of MSA patients showed positive seeding activity
				Maximum ThT fluorescence significantly distinguished MSA from HC (AUC = 0.97) and PD/DLB (AUC = 0.93)
				The fluorescence plateau occurred significantly earlier in MSA than PD/DLB
	Shahnawaz et al. [45]	75 MSA, 94 PD, 56 HC	PMCA	86.7% (66/75) of MSA patients showed positive seeding activity
				The fluorescence plateau occurred significantly earlier and was lower in MSA compared to PD
				The biochemical and morphological features of α-syn aggregates were different between MSA and PD
	Poggiolini et al. [47]	24 MSA, 74 PD, 45 iRBD, 55 HC	RT-QuIC	75.0% (18/24) of MSA patients showed positive seeding activity
				RT-QuIC parameters were associated with UMSARS change
	Rossi et al. [48]	33 MSA, 48 DLB, 60 AD, 31 PSP/CBS, 71 PD, 18 iRBD, 28 PAF, 143 HC	RT-QuIC	Pathologic seeding activity was seldom found in MSA
	Quadalti et al. [49]	68 MSA, 116 PD, 52 PSP, 34 HC	RT-QuIC	Only 4.4% (3/68) of MSA patients showed positive seeding activity
Saliva	Luan et al. [50]	18 MSA, 75 PD, 36HC	RT-QuIC	61.1% (11/18) of MSA patients showed positive seeding activity
				The fluorescence plateau significantly distinguished MSA from HC (AUC = 0.8194)
				The lag phase was significantly longer in MSA than in PD
Skin	Wang et al. [41]	3 MSA, 47 PD, 7 LBD, 17 AD, 8 PSP, 5 CBD, 43 HCs (autopsy)	RT-QuIC, PMCA	66.7% (2/3) of MSA patients showed positive seeding activity
	Donadio et al. [140]	8 MSA, 17 PD, 5 DLB, 3 PAF	RT-QuIC	Seeding activity from patients with synucleinopathies was higher than that of non-synucleinopathies
Olfactory mucosa	De Luca et al. [146]	11 MSA, 18 PD, 6 CBD, 12 PSP	RT-QuIC	81.8% (9/11) of MSA patients showed positive seeding activity
				The biochemical and morphological features of α-syn aggregates were different between MSA and PD
	Bargar et al. [147]	30 MSA, 13 PD, 11 HC	RT-QuIC	90% (18/20) of MSA-P and only 10% (1/10) of MSA-C patients showed positive seeding activity
				Seeding activity positively correlated with rigidity and postural instability
	De Luca et al. [148]	2 MSA, 2 PD, 1 HC	RT-QuIC	100% (2/2) of MSA patients showed positive seeding activity
				The biochemical and morphological features of α-syn aggregates were different between MSA and PD

*AD* Alzheimer disease, *CBD* corticobasal degeneration, *CBS* corticobasal syndrome, *DLB* dementia with Lewy bodies, *HC* healthy control, *iRBD* Idiopathic REM sleep behaviour disorder, *MSA* multiple system atrophy, *PAF* pure autonomic failure, *PD* Parkinson’s disease, *PMCA* protein misfolding cyclic amplification, *PSP* progressive supranuclear palsy, *RT-QuIC* real-time quaking-induced conversion

Due to the invasive nature of CSF collection, more easily accessible fluids are in need to investigate the seeding activity of pathological α-syn. Only one study assessed the use of salivary samples and found that salivary α-syn RT-QuIC assay had a detection sensitivity of 61.1% in MSA patients [50] (Table 2). Overall, there has been increasing evidence for the potentials of humoral α-syn seeding activity as a reliable biomarker for MSA. Further validation in larger cohorts including various samples using established uniform experimental procedures is necessary.

### Coenzyme Q10 (CoQ10)

CoQ10 is a large, lipid-soluble molecule located at the cell membrane. It is a powerful cellular antioxidant and can transport electrons from complexes I & II to complex III in the mitochondrial electron transport chain. One of the essential enzymes involved in CoQ10 biosynthesis is hydroxbenzoate polyprenyltransferase (CoQ2) encoded by the *COQ2* gene [51]. Previous studies have found that homozygous and compound heterozygous mutations in *COQ2* may be the etiology of familial and sporadic MSA, and several mutation sites in *COQ2* have been identified to be associated with increased susceptibility to MSA. In addition, the level of CoQ10 and its activity have been proven to be decreased in MSA patients with *COQ2* mutation [52–55]. Subsequent studies also showed impaired mitochondrial function and significant reduction of CoQ10 levels in lymphoblastoid cells, fibroblasts and cerebellum of MSA patients [56–58], suggesting the relationship of CoQ10 with MSA pathogenesis.

Only one study has investigated the diagnostic value of CSF CoQ10 levels in MSA patients so far. Compared with HC, PD or PSP patients, the level of CSF CoQ10 in MSA patients was significantly decreased, but did not differ between MSA-C and MSA-P subtypes [59]. The conclusions deserve further consideration due to the small sample size employed in the study. Most of the studies investigating CoQ10 levels in blood revealed lower CoQ10 levels in MSA patients compared to HCs [60–62]. Clinical association analysis results showed a weak negative correlation between plasma CoQ10 levels and UMSARS-II scores in MSA-C patients [60]. None of these studies found significant differences between patients with MSA and PD, nor between different subtypes of MSA (Table 1). Although the conclusions of current studies are consistent, the reliability still needs to be further validated, because the CoQ10 level can be affected by many confounders, including dietary habits, lipoprotein levels, hyperthyroidism and drugs [61].

### Neurofilament light protein (NfL)

Neurofilament is a neuronal cytoskeletal protein, which is released upon axonal damage, into the extracellular space and subsequently to CSF and blood. NfL is the smallest subunit of neurofilament protein and a marker for central neuronal damage, particularly sensitive to axonal destruction of long fibre tracts [63, 64]. Since axonal injury is a specific pathogenic process in neurodegenerative diseases, theoretically, NfL may serve as a biomarker for neurodegenerative diseases. The sensitivity of traditional enzyme linked immunosorbent assay (ELISA) to detect blood NfL is limited due to the low concentration (picograms per milliliter). In addition, repeated detection of CSF is inconvenient, even if the concentration of CSF NfL is relatively high. Recently, the development of single-molecule array (Simoa) technique with ultra-sensitivity has allowed quantification of the analyte at levels < 1 pg/ml, paving the way for evaluating NfL levels in both central and peripheral fluids [63, 65]. NfL has been proposed as a sensitive diagnostic or prognostic marker for various neurodegenerative diseases, including PD, Alzheimer’s disease (AD), spinocerebellar ataxia type 3 (SCA3), amyotrophic lateral sclerosis (ALS) and Huntington’s disease (HD) [66–70]. Likewise, NfL may also be a potential biomarker for MSA since neuronal damage is a crucial aspect of MSA pathogenesis as well (Fig. 2).

Among the studies of CSF NfL, both ELISA and Simoa technique showed markedly elevated CSF NfL levels in MSA patients compared to HC and PD with satisfactory discrimination [16, 21, 71–74]. Meanwhile, the high correlation between CSF and blood NfL levels has been acknowledged since NfL could penetrate through the blood–brain barrier [73, 75, 76]. Compared with HCs and PD patients, MSA patients show significantly increased serum and plasma levels of NfL, both of which display strong differentiating power with an AUC > 0.9 against HC and > 0.8 against PD [66, 73, 74]. In addition, serum NfL is able to differentiate MSA-C from SAOA and SCA according to a pilot study, with significantly higher levels in MSA patients [64]. These findings suggest that humoral NfL might serve as a valuable biomarker in diagnosis and differential diagnosis of MSA. However, studies on its performance in discriminative diagnosis of MSA from other neurodegenerative diseases are needed (Table 1). As for the association with disease progression, previous studies consistently showed that blood NfL levels are significantly correlated with disease severity and the baseline concentration is a good predictor for MSA progression [73, 74, 77]. These results suggest that the blood NfL is a reliable biomarker for monitoring the disease severity and the therapeutic effect on MSA.

### YKL-40

YKL-40, also known as chitinase-3-like-1, is a member of the 18-glycosyl hydrolase family and extensively expressed in various cells such as chondrocytes, synovial cells, neutrophils, macrophages, astrocytes and microglia. Previous studies have verified the participation of YKL-40 in inflammation. Since neuroinflammation with activation of microglia and astrocytes is an important pathogenic process in MSA, YKL-40 may be a potential biomarker of MSA [16, 78] (Fig. 2). However, the conclusions of published studies are inconsistent. Two studies using CSF samples showed no significant difference between MSA patients and HCs [79, 80], while another study demonstrated significantly higher CSF YKL-40 levels in MSA patients than in HCs [16]. A meta-analysis based on the above two studies [79, 80] found no significant difference of CSF YKL-40 levels between MSA patients and HCs [81]. There is also inconsistency for the comparison of MSA vs PD patients. Two studies found that the level of YKL-40 in the CSF of MSA patients was significantly higher than that of PD patients [79, 80], but another study found no significant difference [16]. Only one study compared serum YKL-40 levels in MSA patients with HCs and PD patients, and no significant difference was found [80] (Table 1). Based on the existing findings, YKL-40 may not be a reliable biomarker for MSA, despite the limited number of relevant studies.

### Glial fibrillary acidic protein (GFAP)

GFAP is another marker of glial cell activation and plays an important role in the communication between glial cells and Purkinje cells. Because the activation of glial cells is a vital pathological process in MSA, GFAP may serve as a potential biomarker for MSA (Fig. 2). When comparing MSA patients with HCs, current studies all show that CSF concentrations of GFAP tend to be higher in MSA patients, but without statistical significance [82–84]. When comparing MSA with PD, studies have reported higher or similar CSF GFAP levels in MSA [83, 84] (Table 1). In addition, a meta-analysis concluded that the CSF levels of GFAP in MSA patients are significantly higher than that in HCs; however, this meta-analysis included limited number of studies [21]. In brief, the use of GFAP as a biomarker of MSA needs to be further explored.

### Myelin basic protein (MBP)

MBP is the second most abundant protein in the central nervous system (CNS) myelin and is bound to the cytosolic surface of the oligodendrocyte membrane. MBP is essential for the formation of CNS myelin. It also plays vital roles in signaling, interacting with the cytoskeleton, regulating the expression of other myelin proteins and binding polynucleotides in the nucleus [85]. The levels of MBP may be elevated in the CSF of MSA patients since demyelination is an important part of the pathogenesis of MSA [86] (Fig. 2). Researchers have analyzed MBP levels in the CSF and showed significantly increased concentrations of MBP in MSA when compared with HCs and PD [84, 87]. Moreover, one study demonstrated that the CSF MBP could differentiate MSA and PD at early stages with high accuracy [84] (Table 1). Thus, CSF MBP has the potential to be a diagnostic biomarker of MSA. Validation in larger sample cohorts is needed.

### Homocysteine (Hcy)

Hcy is a sulfur-containing amino acid generated during methionine metabolism. Previous studies have indicated that Hcy is involved in the modulation of *N*-methyl-*D*-aspartate (NMDA) receptor, and is associated with neuroinflammation, oxidative stress, neuronal apoptosis, signaling pathway regulation, and mitochondrial dysfunction [88, 89]. These physiological processes play vital roles in the pathogenesis of neurodegenerative diseases, and hyperhomocysteinemia has been proven to increase the risk of AD and PD [90–94]. Besides, a randomized controlled trial showed that the serum level of Hcy is positively correlated with the rate of brain atrophy [95]. Therefore, Hcy may serve as an indicator of neurodegenerative disorders.

Serum Hcy is gaining increasing attention in the field of MSA. Most studies have revealed that the serum levels of Hcy in MSA patients are significantly increased compared with HCs, especially in male patients [96–99]. A meta-analysis based on three studies also showed higher serum Hcy levels in MSA patients than in HCs [81]. When compared with PD patients, a study found that the serum Hcy level in male MSA patients is significantly higher than that in male PD patients, while no significant difference was observed in females [99] (Table 1). In addition, the serum Hcy levels are positively correlated with the Hoehn-Yahr stage, the UMSARS-IV score, and the nonmotor symptoms score burden—cardiovascular, and negatively correlated with the MMSE score of MSA patients, indicating that Hcy is associated with aggravation of motor and non-motor dysfunction in MSA [97, 99]. However, the specificity of Hcy requires further evaluation since it can be involved in the pathophysiology of various human diseases.

### RNA

Transcription of genes is a critical process as it is the driver of protein expression and other cellular activities, which can adapt rapidly to physiological or pathological changes. Thus, transcriptional profiles provide possibilities as promising biomarkers for diseases. Besides messenger RNAs (mRNAs) which account for 1%–2% of human transcriptome, non-coding RNAs, such as microRNAs (miRNAs), long non-coding RNAs (lncRNAs) and circular RNAs (circRNAs), are also receiving increasing attention due to their regulatory functions in transcription, splicing and translation [100, 101]. In addition, the emerging transcriptomics investigating the comprehensive RNA transcripts in a high-throughput manner offers an encouraging approach for transcriptional biomarker discovery [102].

#### mRNAs

The biomarker value of mRNAs has been proven in various neurodegenerative diseases, such as AD and PD where several classifiers consisting of different blood RNA profiles show high distinguishing accuracies [103–106]. For MSA, both microarrays and RNA sequencing have been performed to investigate the differentially expressed genes (DEGs) in brain tissues compared to HCs [107–110]. However, only one study has assessed the transcriptomic landscape in the peripheral blood of MSA patients, which found distinct expression profiles of MSA compared to HC and PD, including DEGs involved in the nervous system development, cytoskeleton, protein modification, etc. Besides, the discrepancy between MSA subtypes has also been revealed, that MSA-P patients possess more DEGs than MSA-C patients when compared to HCs [111]. Another study analyzed the levels of several targeted gene transcripts from nasal fluid cells in MSA and found reduced mRNA levels of parkin and AIMP2 in MSA patients compared to HC [112]. Although none of the studies directly explored the distinguishing accuracy of altered gene transcripts, the existing evidence suggests the biomarker potential of mRNAs for MSA. Thus, more transcriptomics studies of different clinical samples are needed to provide additional evidence for the biomarker value of mRNAs in MSA.

#### MiRNAs

MiRNAs are small single-stranded non-coding RNAs found in almost all eukaryotic cells and several viruses [113]. MiRNAs negatively regulate the expression of target genes by promoting the inhibition and degradation of mRNAs. On the one hand, previous studies have observed that miRNAs are widespread in the CNS and that aberrant expression of miRNAs may cause neurodegeneration by various mechanisms including mitochondrial dysfunction, oxidative stress, autophagy, α-syn accumulation, synaptic transmission and neuroinflammation [114–116]. On the other hand, several disease-related proteins in neurodegenerative disorders have been proven to influence miRNAs [117–119]. Thus, increasing attentions are being paid to miRNAs and their functions in neurodegenerative diseases. MiRNAs are widely distributed in biofluids, highly stable and quantifiable [114, 120–123]. Besides, existing transcriptomics studies have reported aberrant miRNAs in MSA, so miRNAs are considered as promising MSA biomarkers.

In the CSF of MSA patients, several miRNAs have been found to be significantly increased (including miR-184, miR-218-5p, and miR-7-5p) or decreased (including miR-19a, miR-19b, miR-24, and miR-34c) compared with HCs, and a combination of these miRNAs presents an improved diagnostic accuracy. There are also significant differences between MSA and PD patients. MiR-184 and miR-7-5p are much higher while miR-106b-5p and miR-let-7b-5p are lower in MSA compared with PD. However, no combined miRNA panel has been found to discriminate MSA from PD with high accuracy [124, 125]. Among these miRNAs, miR-7-5p is more disease-specific, which has been reported to target α-syn mRNA and subsequently regulate α-syn expression and neuron survival [126].

In the blood, a variety of differentially expressed miRNAs have been found in MSA patients compared with HCs and PD patients, including miR-16-5p, miR-24-3p, miR-7641, miR-191, miR-671-5p and miR-19b-3p [127–132]. A combination of serum miR-141, miR-193a-3p and miR-30c has been verified to well discriminate between MSA and HC with an AUC of 0.895, and a diagnostic panel consisting of five miRNAs (miR-31, miR-141, miR-181c, miR-193a-3p and miR-214) accurately differentiated MSA from PD with an AUC of 0.951 [129]. Besides, the expression levels of plasma miR-671-5p, miR-19b-3p and miR-24-3p showed significant differences between the two subtypes of MSA [127].

In conclusion, existing findings indicate that miRNA transcriptomics might be an ideal biomarker for MSA, but the lack of a uniform detection method has limited its application. In addition, only a small number of MSA patients were included in published studies, so large-cohort studies based on multi-center cooperation may be a future direction to better investigate the biomarker value of miRNAs in MSA patients.

## Tissue biomarkers

### Skin

#### α-Syn

The aberrant aggregation of α-syn is considered as the initiating factor for α-synucleinopathies. Previous studies have proven that pathological α-syn is not restricted to the CNS, but could also be detected in multiple peripheral organs and tissues including skin, salivary glands, sympathetic ganglia, vagus nerve, gastrointestinal tract, genitourinary tract, and heart [133, 134]. Such peripheral pathological changes may be a precursor to central pathology and associated with complex clinical symptoms [41].

Skin biopsy is a relatively safe and highly acceptable way to measure α-syn deposition in the peripheral nervous system [135]. Existing studies demonstrate that pS129-α-syn deposition within dermal nerves could be detected in skin of 67%-86% of MSA patients, but could not be found in HCs [136–138]. The results indicate that cutaneous pS129-α-syn might be a reliable biomarker to help identify MSA from HC. Moreover, the location of pS129-α-syn deposition also provides evidence for the differential diagnosis among α-synucleinopathies. Skin biopsies of MSA patients display a prevalent abnormal pS129-α-syn deposition in somatic sensory fibers, whereas PD and DLB patients mainly show a widespread involvement of autonomic fibers [136–139]. Thus, the specific aggregation of pS129-α-syn in skin somatic sensory fibers may help differentiate MSA from other α-synucleinopathies.

The RT-QuIC technology and PMCA have also been applied to measure pathological α-syn in skin and demonstrated significantly higher aggregation seeding activity in individuals with α-synucleinopathies than non-synucleinopathies and HC [41, 140]. However, limited investigations have been conducted in MSA. Only one study included three MSA autopsy skin samples and two of them showed positive seeding activity [41]. Therefore, the seeding activity of α-syn in skin needs to be validated in more cases including both postmortem and antemortem samples. Moreover, the study found variable sensitivity and specificity of RT-QuIC in different body regions, with posterior cervical region exhibiting higher and earlier α-syn seeding activity than abdominal region or leg [41] (Table 2). Thus, standardized procedure and uniform sampling region should be taken into account when detecting cutaneous α-syn.

#### Peripheral skin innervation

Autonomic dysfunction is an important clinical manifestation of MSA. However, autonomic impairment such as orthostatic hypotension, sphincter dysfunction and constipation is also commonly observed in PD and pure autonomic failure (PAF) patients and is not sufficient to distinguish between these disorders [141]. Recently, some scholars have explored the dermal innervations and speculated that the denervation of peripheral nerves may be a useful marker for differentiating among MSA, PD and PAF. Epidermal nerve fiber density (ENFD) reflects the number of unmyelinated fibers per linear millimetre of epidermis. Previous studies have found a pronounced decrease of ENFD in these three disorders, but ENFD is much lower in patients with PD or PAF compared with MSA [136, 139, 142–144]. Besides, studies also revealed different skin autonomic innervations among MSA, PD and PAF. Though distal autonomic innervation of the sweat glands in MSA patents is slightly decreased when compared with HCs, it is relatively preserved in MSA compared to PD and PAF, particularly for the cholinergic and adrenergic autonomic innervation [136, 142]. Accordingly, peripheral skin innervation is helpful for differential diagnosis of MSA, but the diagnostic utility needs further verification due to the limited investigation.

### Olfactory mucosa (OM)

Olfactory dysfunction is a common feature of α-synucleinopathies and α-syn is reported to be more abundant synuclein compared with β- and γ-synuclein in OM [145]. In contrast to CSF, OM samples can be collected non-invasively and repeatedly. Thus, the pathological α-syn in OM may offer as a promising biomarker.

RT-QuIC analysis of OM samples from MSA and PD patients showed higher aggregation seeding activity than non-synucleinopathies and HC [146–148]. Besides, the biochemical and morphological properties of α-syn fibrils are significantly different between PD and MSA [146, 148]. A study further revealed that the seeding activity was present in 90% MSA-P patients whereas no seeding activity was found in almost all MSA-C OM samples [147]. The results suggest that α-syn aggregation seeding activity in OM may be a promising biomarker for discriminating between these two subtypes. Moreover, the seeding activity of α-syn in OM has been reported to be positively related with rigidity and postural instability and shows a tendency of inverse association with disease duration [147] (Table 2). However, whether COVID-19 infection might influence the properties of the OM samples and then alter the results of α-syn seeding activity is unknown, so additional investigations in post-infection population will be needed.

## Gut microbiota

Gut microbiota refers to the microorganisms residing in the human digestive tract, including bacteria, fungi, viruses and bacteriophage. The number of gut microorganisms is between 10^13^ and 10^14^ orders of magnitude, with a range of 500 to 1000 species [149]. It has been reported that gut microbiota plays important roles in regulating immunity, digestion, intestinal endocrine, neurological signaling, and drug and toxin metabolism of the host, as well as producing numerous compounds affecting the physiological status of the host [150]. In particular, the proposed microbiome-gut-brain axis suggests that gut microbiota may participate in the homeostasis and diseases of CNS through immune, neurological and neuroendocrinal pathways [151, 152]. Therefore, the disorganized gut microbiota has been considered as a potential therapeutic target and a possible biomarker for neurodegenerative disorders, such as AD, PD and multiple sclerosis (MS) [153–157].

At present, two methods are mainly used for the detection of gut microbiota: marker-gene studies such as 16S rDNA sequencing and multi-omics technology such as metagenomic sequencing. Both methods have been applied in MSA patients. Increased abundance of putative pro-inflammatory bacteria such as *Bacteroides* has been reported in MSA patients compared to HC, while the abundance of putative anti-inflammatory bacteria such as *Ruminococcaceae* and *Coprobacillaceae* is lower in MSA [158–160]. One study revealed significantly different abundance of fecal and blood microbiota between MSA patients and HC, and that a combination of six genera was predictive of MSA with an AUC of 0.853. In addition, a combined model of five genera from feces and blood was able to differentiate between two subtypes of MSA with an AUC of 0.898 [161]. Another study constructed a set of 25 gut microbial gene markers to discriminate MSA from PD patients with an AUC of 0.831 [155]. Gut microbiota dysbiosis provides a new direction for searching MSA biomarkers. However, due to the limited sample size and inherent discrepancy between 16S rDNA and metagenomic sequencing, the reliability of gut microbiota as a biomarker for MSA needs further exploration. Besides, targeted validation and multi-omics technology are required for obtaining more detailed information of microbiotic function in MSA patients.

## Imaging biomarkers

### Magnetic resonance imaging (MRI)

Morphological MRI is an important auxiliary tool in the clinical diagnosis of MSA. Typical imaging features in brain MRI, such as the “hot-cross bun” sign and the “putaminal slit” sign, are clinically suggestive of MSA, which are recommended in the second consensus statement on the diagnosis of MSA [162, 163]. In clinically or neuropathologically diagnosed cases, the “hot-cross bun” sign has almost 100% specificity for MSA against HC, PD, PSP and CBD, but the sensitivity is much lower (around 50%) [162, 164–166]. In the new Movement Disorder Society (MDS) criteria for the diagnosis of MSA, the “hot-cross bun” sign is still recommended as a supportive biomarker for MSA. However, its diagnostic specificity should be considered with caution when distinguishing MSA from non-degenerative parkinsonism or SCA [4]. As for the “putaminal slit” sign, high specificity (around 90%) and low sensitivity (around 30%) have been reported in differentiating MSA from PD, but the differential diagnostic potential in separating MSA from PSP is limited [162, 164, 165]. In addition, the “putaminal slit” sign is a common feature on 3.0 T MRI images of HC [167]. Therefore, the clinical value of this sign appears to be limited and it is omitted from the new MDS criteria for the diagnosis of MSA [4]. Based on the limitation of conventional imaging biomarkers, more advanced imaging techniques are needed for the development of reliable MSA biomarkers.

Recently, researchers have applied multimodal imaging and machine learning techniques to systematically evaluate MRI abnormalities in MSA patients. The machine learning approach based on volumetry of regions including putamen and infratentorial regions, has been shown to accurately classify MSA, PD and PSP with an accuracy of about 97% [168–172]. In addition to volumetric parameters, other parameters such as fractional anisotropy, mean diffusivity and iron deposition in multimodal imaging approach are also helpful in the diagnosis of MSA [173–179]. A logistic regression model based on fractional anisotropy in the cerebellum, brain stem and bilateral superior corona radiata, together with the mean diffusivity in the right superior frontal gyrus, has been shown to discriminate MSA from PD patients with an accuracy  > 95% [173]. In general, multimodal MRI parameters and artificial intelligence analysis have the potential to further improve the performance of MRI in the diagnosis of MSA.

### Positron emission tomography (PET)

Molecular imaging based on hypometabolism, decreased activity of dopaminergic and cholinergic systems and neuroinflammation has also been used in MSA patients [5]. ^18^F-FDG PET has revealed reduced glucose metabolic activity in the frontal cortex, striatum, cerebellum, and brainstem of MSA patients [180–184]. ^18^F-dopa, ^18^F-FP-CIT, and ^11^C-DTBZ PET which assess the function of dopaminergic system, have shown significantly decreased activity in the putamen, caudate nucleus, ventral striatum, globus pallidus externa and red nucleus in MSA patients [181, 185]. ^11^C-PMP PET which assesses the activity of cholinergic systems, revealed reduction of brain acetylcholinesterase activities in the thalamus and cerebellum in MSA-C patients, while significant decreases in cortical and subcortical cholinergic activity are present in MSA-P patients [186, 187].

Recently, PET using a novel radiotracer ^11^C-UCB‐J that binds to synaptic vesicle protein 2A (SV2A), a synapse-specific protein, has shown high sensitivity in various neurodegenerative diseases, including PD, AD, DLB, PSP [188–191]. The SV2A-targeting PET can also assist the diagnosis of MSA due to the neurodegenerative pathology in MSA. However, since decreased synaptic density is widely present among neurodegenerative diseases, comparative analysis of its performance on different MSA-mimicking disorders is a necessary direction for future research.

For α-synucleinopathies, developing α-syn-specific PET technique is promising as it offers in vivo monitoring of development of α‐syn pathologies and directly reflects the effects of anti-synuclein therapies in living patients compared with other imaging techniques [192]. Moreover, the conformation and distribution of α-syn aggregates in MSA patients are different from those in other synucleinopathies [45, 193], indicating the potential advantages of disease-specific PET ligands in differentiating these diseases. Up to now, nearly 40 different ligands have been developed for α-syn [194, 195], among which more than 20 compounds have been radiolabeled and evaluated in animal models [196–201] or postmortem tissues of patients [202–209]. The PET ligand [^11^C]PBB3 shows distinct affinity to α-syn pathology in different synucleinopathies [204]. Even more encouraging, PET scans with two α-syn ligands ^11^C-BF-227 and ^18^F‐SPAL‐T‐06 in living MSA patients show high distribution in brain regions coincident with the predominant distributions of GCIs, compared with HCs [205, 210]. However, the current radiotracers are still not able to overcome the disadvantages of low values of brain uptake, affinity, selectivity and disease-specificity. Thus, preclinical studies of α-syn-specific PET need to be further conducted before clinical application.

## Conclusions and future perspectives

The clinical diagnosis, severity monitoring and prognosis for MSA are quite challenging, which also restrict the development of novel disease-modifying strategies. Significant efforts have been made to explore biomarkers for MSA in recent years. Here, we comprehensively review the latest progress on the multidimensional biomarkers for MSA, including biomarkers in fluids and tissues, gut microbiota and imaging biomarkers (Fig. 1). Among these biomarkers, α-syn in the CSF, blood and tissues is the most focused target, although inconsistency exists among studies on the exact levels of α-syn. The SAAs targeting the CSF or tissue α-syn are a great step forward and show high differentiating accuracy, including in distinguishing among α-synucleinopathies in particular. The different conformational strains and reaction kinetics of α-syn among α-synucleinopathies might underlie this differentiating accuracy [211]. Exploring the seeding activity of α-syn in other samples such as the saliva may be an alternative since lumbar puncturing and tissue biopsy are a challenge for clinical extension. Furthermore, developing PET imaging ligands to noninvasively detect original α-syn pathology facilitates in vivo studies of the distinct conformations and regional spread of α-syn pathology among α-synucleinopathies, as well as exploration of targets for anti-syn therapies [192]. In addition, NfL levels in fluids have been used as an outcome measure to evaluate the efficacy of drug trials in MSA (NCT05104476, NCT05109091), based on their significant correlation with disease severity. In addition, multimodal imaging combined with machine learning techniques is also a breakthrough in this field.

Although significant progress has been made on biomarker discovery for MSA, there are still great gaps in the application of these biomarkers in clinic. Several reasons may explain the challenges in this field. Since autopsy confirmation is the only gold standard for definite diagnosis and previous studies revealed that the antemortem diagnostic accuracy is only about 62% for MSA [6], the presence of misdiagnosis in current biomarker studies limits the accuracy of conclusions, which might underlie the discrepancy among different studies. Besides, the limited sample size due to the low prevalence of MSA also partially accounts for the inconsistency among studies. Furthermore, the intrinsic heterogeneity between two MSA subtypes sets up obstacles for biomarker identification. Finally, the lack of standard methodologies may be another important reason why results are not always reproducible in this field.

For future explorations, large multi-center cohort studies are necessary. Validation in neuropathologically established MSA cohorts and explorations between different MSA subtypes are needed. Furthermore, unified standards and methodologies are also mandatory to improve the replicability of biomarkers for MSA. In recent years, progresses in high-throughput technologies and artificial intelligence have provided new opportunities for exploring disease biomarkers [212]. Integrating multi-omics data through artificial intelligence technology, including genomics, transcriptomics, proteomics, metabolomics, metagenomics, and radiomics, is able to draw a multi-omics characteristic spectrum of MSA patients and develop a multi-dimensional biomarker system. Constructing diagnostic or predictive panels through the combination of multidimensional biomarkers, based on the novel techniques and artificial intelligence, is quite promising for this field.

In conclusion, great efforts have been made in the field of multidimensional biomarkers for MSA and several potential biomarkers have been identified in the past years. However, there are still quite a few challenges before implementation of these biomarkers in clinic. Applying advanced detection techniques such as SAAs and multi-omics analysis in large-sample cohorts including neuropathologically established MSA cases, will pave the way for exploration of MSA biomarkers.

## Data Availability

Not applicable.

## Abbreviations

α-syn: α-Synuclein
AD: Alzheimer's disease
ALS: Amyotrophic lateral sclerosis
AUC: Area under the curve
CBD: Corticobasal degeneration
CNS: Central nervous system
CoQ10: Coenzyme Q10
CSF: Cerebrospinal fluid
DEG: Differentially expressed gene
DLB: Dementia with Lewy bodies
ENFD: Epidermal nerve fiber density
GCI: Glial cytoplasmic inclusion
GFAP: Glial fibrillary acidic protein
HC: Healthy controls
Hcy: Homocysteine
HD: Huntington's disease
MBP: Myelin basic protein
MRI: Magnetic resonance imaging
MS: Multiple sclerosis
MSA: Multiple system atrophy
NfL: Neurofilament light protein
OM: Olfactory mucosa
PAF: Pure autonomic failure
PD: Parkinson's disease
PET: Positron emission tomography
PMCA: Protein-misfolding cyclic amplification
PSP: Progressive supranuclear palsy
RBCs: Red blood cells
RT-QuIC: Real-time quaking-induced conversion
SAA: Seeding aggregation assay
SAOA: Sporadic adult-onset ataxia of unknown aetiology
SCA: Spinocerebellar ataxia
Simoa: Single-molecule array
SV2A: Synaptic vesicle protein 2A
